# Associação entre Atividade Física no Tempo Livre e HDL-C em Participantes do Elsa-Brasil: Existem Diferenças entre Homens e Mulheres no Efeito Dose-Resposta?

**DOI:** 10.36660/abc.20200438

**Published:** 2021-06-25

**Authors:** Francisco José Gondim Pitanga, Rosane Harter Griep, Maria da Conceição Almeida, Maria de Jesus Mendes da Fonseca, Andreia Rios de Souza, Raiane de Carvalho Silva, Sheila Maria Alvim Matos

**Affiliations:** 1 Programa de Pós-Graduação em Ciências da Reabilitação Universidade Federal da Bahia Salvador BA Brasil Programa de Pós-Graduação em Ciências da Reabilitação - Universidade Federal da Bahia, Salvador, BA - Brasil; 2 Laboratório de Educação em Meio Ambiente e Saúde Instituto Oswaldo Cruz Fiocruz Rio de Janeiro RJ Brasil Laboratório de Educação em Meio Ambiente e Saúde, Instituto Oswaldo Cruz, Fiocruz, Rio de Janeiro, RJ - Brasil; 3 Instituto Gonçalo Moniz Salvador BA Brasil Instituto Gonçalo Moniz, Salvador, BA - Brasil; 4 Escola Nacional de Saúde Pública Fiocruz Rio de Janeiro RJ Brasil Escola Nacional de Saúde Pública (Fiocruz), Rio de Janeiro, RJ - Brasil; 5 Universidade Federal da Bahia Salvador BA Brasil Universidade Federal da Bahia, Salvador, BA - Brasil; 6 Instituto de Saúde Coletiva Universidade Federal da Bahia Salvador BA Brasil Instituto de Saúde Coletiva - Universidade Federal da Bahia, Salvador, BA - Brasil

**Keywords:** Doenças Cardiovasculares, Exercício, Atividade Física, Estudos Transversais, HDL-Colesterol, Epidemiologia, Caminhada

## Abstract

**Fundamento:**

Níveis elevados de lipoproteína de alta densidade (HDL-C) podem ter efeitos positivos para proteção de agravos cardiovasculares, e prática regular de atividade física no tempo livre (AFTL) tem sido associada ao seu aumento.

**Objetivo:**

Verificar a existência de possíveis diferenças entre homens e mulheres no efeito dose-resposta na associação entre AFTL e HDL-C.

**Métodos:**

Estudo transversal com dados de 13,931 participantes de ambos os sexos (7,607 mulheres) do Estudo Longitudinal da Saúde do Adulto (ELSA-Brasil). A AFTL foi mensurada por meio do International Physical Activity Questionnary (IPAQ) e classificada em quatro categorias: inativos, pouco ativos, ativos e muito ativos. O poder discriminatório da AFTL para HDL-C elevado, nas diferentes intensidades analisadas (caminhada, atividade física moderada e atividade física vigorosa) foi testado por meio das curvas ROC. As associações, ajustadas por variáveis de confundimento entre AFTL e HDL-C foram analisadas por meio de regressão logística, estimando-se a odds ratio (OR) com intervalo de confiança (IC) de 95%.

**Resultados:**

Observou-se associação positiva, com efeito dose-resposta, entre AFTL e HDL-C tanto em homens quanto em mulheres. Com relação à intensidade, apenas a atividade física vigorosa discriminou o HDL-C elevado em homens, enquanto tanto a atividade física de caminhada quanto a moderada e a vigorosa discriminaram o HDL-C elevado em mulheres.

**Conclusão:**

A AFTL apresenta associação positiva com gradiente dose-resposta com HDL-C; no entanto, entre os homens, a associação não é observada para aqueles classificados como pouco ativos fisicamente. Nas mulheres, tanto a intensidade da caminhada quanto a atividade física moderada ou vigorosa podem discriminar níveis de HDL-C mais altos; já nos homens, essa relação é observada apenas na intensidade vigorosa.

## Introdução

A prática regular da atividade física (AF) apresenta associação positiva com o aumento dos níveis de lipoproteína de alta densidade (HDL-C) e redução dos níveis de triglicérides, com alterações pouco consistentes em relação aos níveis de lipoproteína de baixa densidade (LDL–c).^1^ Ademais, maiores níveis de HDL-C podem apresentar benefícios na proteção contra doença arterial coronariana; entretanto, estudos mais recentes de randomização mendeliana têm sugerido o HDL-C mais como um marcador de risco cardiovascular do que propriamente um fator de risco.^2 , 3^

O aumento do HDL-C em função da prática de AF pode ser explicado na medida em que, durante a mesma, há aumento da atividade da lipoproteína lipase (LPL), acelerando, assim, a quebra das lipoproteínas ricas em triglicérides. Com isso, gera-se a transferência de colesterol, fosfolipídio e proteínas para as partículas nascentes do HDL-C.^4^ Além disso, em indivíduos mais ativos fisicamente, o tempo médio de vida do HDL-C pode alcançar 6 a 7 dias, enquanto, nos inativos fisicamente, é de apenas 3 a 4 dias.^5^

Apesar dessas evidências, a intensidade ideal de AF para proporcionar o aumento do HDL-C precisa ser melhor esclarecida, principalmente com ênfase em possíveis diferenças entre homens e mulheres demonstrada em estudos prévios sobre AF e saúde cardiometabólica.^6^

Em recente pesquisa feita em participantes do Estudo Longitudinal de Saúde do Adulto (ELSA-Brasil), os resultados mostraram que existe associação entre AF e maiores níveis de HDL-C, tanto em homens quanto em mulheres, Além disso, observou-se que a prática de AF em intensidade vigorosa associava-se com mudanças mais positivas nos perfis lipídicos do que apenas a duração da prática da AF. No entanto, nesse estudo, não foram analisadas possíveis diferenças entre homens e mulheres no que diz respeito à quantidade (tempo e duração) de AF.^7^

O conhecimento da existência de efeito dose-resposta entre a AF e o aumento do HDL-C irá agregar a produção de novas informações e pesquisas sobre o tema, considerando que as políticas públicas de promoção da AF poderão usar essas informações como base para novas propostas de intervenção. Além disso, apesar de a associação entre AF e os níveis de HDL-C ser conhecida, estudos que demonstrem o efeito dose-resposta no que diz respeito a possíveis diferenças entre homens e mulheres são escassos na literatura consultada sobre o assunto. Pesquisas que reafirmam a associação entre AF e HDL-C ainda são de suma importância para um melhor entendimento dessa associação, principalmente ao se analisar as possíveis diferenças entre sexos nesses achados.

O objetivo do estudo foi verificar a existência de diferenças entre homens e mulheres no efeito dose-resposta na associação entre AF e HDL-C entre participantes do ELSA-Brasil.

## Métodos

### População e amostra

O ELSA-Brasil é um estudo de coorte de 15.105 servidores públicos ativos ou aposentados na faixa etária de 35 a 74 anos de seis instituições de nível superior, localizadas nas cidades de Salvador, Vitória, Belo Horizonte, Rio de Janeiro, São Paulo e Porto Alegre, que tem como principal objetivo investigar a incidência e a progressão de diabetes e doenças cardiovasculares e seus fatores associados (biológicos, comportamentais, ambientais, ocupacionais, psicológicos e sociais), cujos detalhes metodológicos foram descritos previamente.^8 , 9^ Até o momento, já foram coletadas informações sobre os participantes em três oportunidades: 1ª onda, de 2008 até 2010; 2ª onda, de 2012 até 2014; e 3ª onda, de 2016 até 2018. Para o presente estudo, foram selecionados todos os participantes da Onda 2 (2012-2014) que responderam aos questionários sobre AF e com dados completos relativos às variáveis envolvidas na análise, totalizando 13.931 participantes (7.607 mulheres).

O ELSA-Brasil foi aprovado pela Comissão Nacional de Ética em Pesquisa (CONEP) e em todos os Comitês de Ética em Pesquisa dos seis centros de investigações envolvidos. Todos os participantes assinaram o termo de consentimento livre e esclarecido, sendo garantidos o sigilo e a confidencialidade dos dados.

### Produção de dados

Os dados foram coletados por uma equipe de entrevistadores e aferidores treinados e certificados por um comitê de controle de qualidade,^9^ capacitados a executar o protocolo do estudo em qualquer Centro de Investigação ELSA-Brasil. Realizaram-se entrevistas face a face na aplicação dos blocos de questionários para obtenção das informações sobre idade, escolaridade, renda familiar e tabagismo. Além disso, foram coletadas informações sobre medidas antropométricas de peso, estatura e circunferência da cintura. O peso corporal foi obtido pela manhã, após 8 a 12 horas de jejum e com o participante sem sapatos e com roupas leves. Foi usada balança eletrônica Toledo^®^, com capacidade para até 200kg. Para medida da estatura, foi usado estadiômetro da marca SECA^®^, com o participante posicionado de pé e seguindo rigorosamente o plano de Frankfurt. A obesidade foi identificada por meio do índice de massa corporal (IMC), aplicando-se a equação IMC = peso (kg)/altura(m)^2^, adotando-se o seguinte ponto de corte; obesidade = 0 se IMC <30,0 e obesidade = 1 se IMC ≥30,0.

### Avaliação da atividade física

Para identificação e quantificação da AF, foi utilizado o International Physical Activity Questionnary (IPAQ), versão longa, que é constituído de questões relativas à frequência e à duração de atividades físicas (caminhada, AF moderada e AF vigorosa) desenvolvidas no trabalho, no deslocamento, nas atividades domésticas e no tempo livre.^10^ No ELSA-Brasil, apenas os domínios do tempo livre e do deslocamento foram avaliados. A AF foi mensurada em minutos/semana por meio da multiplicação da frequência semanal pela duração de cada uma das atividades realizadas. Para efeito desse estudo, utilizou-se apenas a atividade física no tempo livre (AFTL), com a seguinte categorização: 0 = inativo fisicamente (<10 minutos de qualquer AF na semana); 1 = pouco ativo (de 10 minutos a menos de 150 minutos de caminhada/moderada por semana e/ou de 10 minutos a menos que 60 minutos de atividades vigorosas por semana e/ou de 10 minutos a menos que 150 minutos por semana de qualquer combinação de caminhada, moderada e vigorosa); 2 = fisicamente ativo (≥150 minutos de caminhada/atividade moderada por semana e/ou ≥60 minutos de atividades vigorosas por semana e/ou ≥150 minutos por semana de qualquer combinação de caminhada, moderada e vigorosa); 3 = muito ativo (≥150 minutos de atividades vigorosas por semana ou ≥60 minutos de atividades vigorosas por semana + 150 minutos por semana de qualquer combinação entre caminhada e moderada). Para as análises dicotomizadas, foram considerados como insuficientemente ativos aqueles classificados como inativos fisicamente e pouco ativos, e como ativos aqueles classificados como fisicamente ativos e muito ativos.

### Avaliação do HDL-C

A coleta de sangue foi realizada após 12 horas de jejum durante a noite e centrifugadas e armazenadas em criotubos em temperatura de –80°C nos próprios centros de investigação. Como forma de garantir a qualidade e a padronização dos resultados, todas as amostras foram enviadas para processamento e análise em um laboratório central do ELSA-Brasil localizado na cidade de São Paulo. Os valores de HDL-C em mg/dL foram determinados por um método colorimétrico homogêneo usando um equipamento ADVIA 1200 Siemens^®^.^7^ O HDL-C foi dicotomizado em <60mg/dL e ≥60mg/dL (HDL-C elevado).

### Covariáveis

Para as covariáveis, adotou-se a seguinte categorização: idade = 0, se entre 34 e 50 anos; idade = 1, se entre 51 e 60 anos; e idade = 2, se >60 anos. Para o grau de instrução, foram estabelecidos quatro estratos: 0 = fundamental incompleto; 1 = fundamental completo; 2 = médio completo; e 3 = superior completo/pós-graduação. A renda familiar foi categorizada em: até 2 salários mínimos = 0; de 2 até 8 salários mínimos = 1; de 8 até 18 salários mínimos = 2; e acima de 18 salários mínimos = 3. O tabagismo atual foi categorizado em não = 0 e sim = 1.

### Análise dos dados

As medidas descritivas (proporções) foram calculadas para todas as variáveis categorizadas. As análises foram estratificadas por sexo *a priori* e comparadas por meio do teste Qui-quadrado. Para testar o poder discriminatório das diferentes intensidades de AFTL (caminhada, AF moderada e AF vigorosa) para HDL-C elevado, foram utilizadas curvas ROC (Receiver Operating Characteristic).^11^ Utilizou-se intervalo de confiança (IC) a 95%. As associações foram analisadas por meio de regressão logística. Foram consideradas como potenciais confundidoras ou modificadoras de efeito as seguintes variáveis: idade, obesidade, tabagismo no momento da entrevista, renda familiar e escolaridade. Foram selecionadas para modelagem as variáveis que, na etapa bivariada (matriz tetracórica), apresentaram na avaliação simultânea p ≤0,05 e coeficiente de correlação rho <0,60.

A verificação da modificação do efeito foi feita por meio de estratificação com a observação das medidas pontuais estrato-específicas e os seus intervalos de confiança. Quando a medida pontual de um fator, em determinado estrato específico, não estava contida no intervalo de confiança do outro fator no mesmo estrato, isso indicava modificação de efeito. A análise das possíveis variáveis de confundimento foi realizada pelo procedimento *backward* , por meio de regressão logística. A análise começa com o modelo completo, seguido pela remoção um a um dos potenciais confundidores. Quando observada modificação igual ou superior a 10% na associação pontual entre AFTL e HDL-C, a variável é considerada confundidora.^12^ No processo de modelagem, não foram identificadas variáveis modificadoras de efeito, e a variável idade foi identificada como confundidora. Portanto, o melhor modelo para análise da associação entre AFTL e HDL-C foi aquele ajustado por idade. Foi também analisado o efeito dose-resposta na associação entre AFTL e HDL-C. Para esta análise, foram criadas variáveis DUMMIES para comparação entre o grupo de referência (inativo fisicamente) e cada um dos outros estratos da variável AF (insuficientemente ativo, ativo e muito ativo). Foi utilizado o teste de Mantel-Haenszel para testar a homogeneidade dos valores da *odds ratio* (OR) entre os estratos de cada variável com nível de significância de 5%. O intervalo de confiança foi estabelecido em 95%. Empregou-se o programa estatístico STATA^®^ versão 12.0.

## Resultados

Um total de 6.324 homens e 7.607 mulheres foi incluído na análise. As características da amostra estão apresentadas na Tabela 1 . A média de idade entre as mulheres é de 55,7±8,8 e entre os homens de 55,7±9,2. Observa-se também que os homens têm maior renda familiar, são mais tabagistas e mais ativos no tempo livre, enquanto as mulheres são mais obesas e apresentam maior proporção de níveis de HDL-C mais elevados. Observa-se ainda que não existem diferenças estatisticamente significativas entre homens e mulheres com relação à idade.

**Tabela 1:** – Características da amostra do estudo

	Homens (n = 6.324)	Mulheres (n = 7.607)	p
**Idade (anos) - n (%)** 34-50 51-60 >60	2.055 (32,5) 2.182 (34,5) 2.087 (33,0)	2.342 (30,8) 2.752 (36,2) 2.511 (33,0)	0,49
**Renda familiar (salário mínimo) – n (%)** Até 2 De 2 até 8 De 8 até 18 Acima de 18	60 (0,9) 2.117 (33,6) 2.462 (39,1) 1.657 (26,3)	84 (1,1) 2.545 (33,6) 3.356 (44,3) 1.583 (20,9)	<0,01
**Escolaridade – n (%)** Fundamental incompleto Fundamental completo Médio completo Superior completo/Pós-graduação	448 (10,2) 482 (10,9) 1.933 (43,9) 1.533 (34,9)	261 (4,5) 390 (6,7) 2.449 (42,3) 2.694 (46,)	<0,01
**Obesidade – n (%)** IMC <30 kg/m^2^ IMC ≥30 kg/m^2^	4.763 (75.3) 1.561 (24,7)	5.308 (69,8) 2.299 (30,2)	<0,01
**Tabagismo atual – n (%)** Não Sim **Atividade física no tempo livre – n (%)** Inativo fisicamente Insuficientemente ativo Ativo Muito ativo **HDL-c elevado** Sim Não	3.290 (52,0) 3.033 (48,0) 2.218 (35,1) 1.089 (17,2) 1.749 (27,6) 1.268 (20,1) 916 (14,5) 5.408 (85,5)	4.863 (63,9) 2.744 (36,1) 3.401 (44,7) 1.342 (17,6) 1.947 (25,6) 917 (12,0) 3.241 (42,7) 4.366 (57,3)	<0,01 <0,01 <0,01

*Valores para homens e mulheres foram comparados por meio do teste Qui-quadrado.*

Na Tabela 2 , pode-se observar o poder discriminatório das diferentes intensidades de AFTL para o aumento do nível de HDL-C. Entre homens, apenas a AF vigorosa apresentou áreas sob a curva ROC estatisticamente significativas, enquanto, entre as mulheres, tanto a caminhada quanto as atividades físicas moderadas e vigorosas apresentaram significância estatística para discriminar o aumento do HDL-C; apesar das áreas sob as curvas ROC não apresentarem valores elevados.

**Tabela 2:** – Áreas sob as curvas ROC para diferentes intensidades de atividade física como discriminador de HDL-c elevado (≥60 mg/dL)

Atividade física no tempo livre	Masculino	Feminino
Caminhada	0,52 (0,50-0,54)	0,52 (0,51-0,53) *
Moderada	0,52 (0,50-0,54)	0,53 (0,53-0,54)*
Vigorosa	0,54 (0,52-0,56)*	0,53 (0,52-0,54)*

ROC: receiver operating characteristic; IC95%: intervalo de confiança a 95%. *Área sob a curva ROC apresentando poder discriminatório IM (Li-IC > 0,50).

A associação entre AFTL e HDL-C em indivíduos do sexo masculino e feminino está demonstrada na Tabela 3 . Observa-se que homens ativos têm 38% de chance de ter níveis mais altos de HDL-C, enquanto, nas mulheres ativas, a chance é de 41%, em comparação aos homens e mulheres inativos fisicamente, respectivamente.

**Tabela 3:** – Associação entre atividade física no tempo livre e HDL-c

Atividade física no tempo livre	Masculino	Feminino
Insuficientemente ativos (Inativos fisicamente e pouco ativos) Ativos (Ativos e muito ativos fisicamente)	1,00 1,38 (1,20-1,59)	1,00 1,41 (1,29-1,55)

* Ajustado por idade

Na Tabela 4 , quando a AF foi classificada em quatro categorias, podemos observar a existência de efeito dose-resposta na associação entre AFTL e HDL-C, tanto em homens quanto em mulheres. Contudo, entre os indivíduos do sexo masculino, a associação foi encontrada apenas naqueles classificados como ativos e muito ativos fisicamente.

**Tabela 4:** – Efeito dose-resposta na associação entre atividade física no tempo livre e HDL-c

Atividade física no tempo livre	Masculino	Feminino
Inativo Pouco ativo Ativo Muito ativo	1,00 1,21 (0,98-1,50) 1,27 (1,05-1,52) 1,77 (1,46-2,15)	1,00 1,15 (1,01-1,31) 1,33 (1,19-1,49) 1,81 (1,56-2,09)

* Ajustado por idade.

## Discussão

O estudo analisou a existência de efeito dose-resposta na associação entre AFTL e o aumento do HDL-C.

É importante salientar que a população do estudo se constitui de uma coorte de voluntários de servidores públicos, que, apesar de não ser representativa da população de modo geral, apresenta um número significativo de participantes em seis capitais brasileiras, fato que representa ponto forte do estudo

Os mecanismos que relacionam a maior intensidade de AF e mudanças lipídicas mais positivas são explicados em função de que, durante a AF, há aumento da atividade da LPL e do catabolismo dos triglicérides que resultam na transferência de colesterol, fosfolipídios e proteínas para as partículas nascentes do HDL-C, aumentado assim sua concentração.^4 , 5^ Além disso, o tempo médio de vida das partículas de HDL-C é maior em indivíduos mais ativos fisicamente, fato que pode contribuir para que o transporte reverso do colesterol possa ser maximizado.^5^

Neste contexto, em pesquisa realizada com participantes do ELSA-Brasil, demonstrou-se que há uma associação benéfica entre os níveis de AF e perfil lipídico favorável para HDL-C e triglicérides, tanto em homens quanto em mulheres. Além disso, observou-se que quanto maior fosse a intensidade da AF, mais positivas seriam as mudanças no perfil lipídico.^7^

Em outro estudo, realizado com indivíduos residentes de Caxias do Sul/RS com mais de 60 anos de idade, foi demonstrado que aqueles que praticavam atividades físicas de intensidade moderada tinham níveis de LDL-c reduzidos e HDL-C aumentados quando comparados com os idosos que tinham baixos níveis de AF.^13^

Segundo Matsudo et al.,^14^ existem poucas evidências a respeito dos mecanismos responsáveis pela redução dos níveis de LDL-c e lipoproteína de muito baixa densidade (VLDL-c). Contudo, a principal razão para elevação do HDL-C é a maior ação da LPL em resposta a exercícios físicos, fato que aumenta o catabolismo dos triglicérides e provoca a transferência de colesterol, fosfolipídios e proteínas para as partículas de HDL-C, aumentando assim a sua concentração.^14^ Outros estudos demonstraram que homens e mulheres ativos fisicamente têm maiores níveis de HDL-C e menores níveis de LDL-c e VLDL-c que seus pares inativos fisicamente,^15^ além de que, em outro trabalho realizado apenas com pessoas do sexo feminino, foi observado que há diferenças significativas nos níveis de HDL-C em mulheres com alta, moderada e baixa aptidão física, e os valores mais altos foram encontrados nas mulheres com níveis altos de aptidão física.^16^

É importante enfatizar também que, no nosso estudo, a intensidade de AFTL foi classificada em caminhada, AF moderada e AF vigorosa. Neste contexto, apesar de as áreas sob as curvas ROC não apresentarem valores elevados, entre os homens, apenas a AF vigorosa discrimina o HDL-C elevado, enquanto, entre as mulheres, tanto a caminhada quanto a AF moderada ou vigorosa discriminam o HDL-C elevado, demonstrando que os homens precisam de AF mais intensa para aumentar os níveis de HDL-C.

Mais um aspecto importante a ser destacado, no presente estudo, é que o efeito dose-resposta entre AFTL e HDL-C tem significância tanto nas mulheres classificadas como pouco ativas quanto nas mulheres classificadas como ativas e muito ativas. Nos homens, por sua vez, a associação é observada apenas naqueles classificados como ativos e muito ativos. Desta forma, é possível compreender que eles têm a necessidade de praticar maior quantidade de AF para que tenham um resultado positivo quanto ao aumento do HDL-C.

Em recente publicação, também com dados do ELSA-Brasil, com objetivo de verificar a associação entre AFTL e AF no deslocamento com escores de risco cardiovascular, foi observado que apenas a AFTL estava inversamente associada com os eventos cardiovasculares, além de que os homens necessitavam de maior quantidade de AF (duração e intensidade), também com efeito dose-resposta, do que as mulheres para que os benefícios fossem alcançados.^17^

Esses resultados podem ser parcialmente explicados pelo fato de que, nos homens, os parâmetros homeostáticos em repouso – tais como frequência cardíaca, pressão arterial, níveis glicêmicos, gasto calórico, entre outros – são mais elevados do que nas mulheres, apesar de que, na pós-menopausa e com o processo do envelhecimento, os valores de pressão arterial podem ser maiores no sexo feminino.^18 , 19^ Assim, para quebrar a homeostase de repouso, os homens precisam de maior quantidade de AF (duração e intensidade) do que as mulheres, para, com isso, fazer com que possam ser ativados os mecanismos que desencadeiam a proteção cardiovascular, tais como redução da pressão arterial, redução dos níveis glicêmicos e de triglicérides, além do aumento do HDL-C (que, no repouso, apresenta menores níveis nos homens.^6^

Uma possível limitação pode ser atribuída à informação sobre AF, pois a mesma foi obtida por meio de questionários que, apesar de ser um instrumento largamente utilizado em estudos nacionais e internacionais, pode apresentar problemas como, por exemplo, o viés de memória. Ademais, é importante ressaltar que, no presente estudo, foi analisada apenas a AFTL, não sendo considerados os outros domínios da AF, como, por exemplo, deslocamento, AF no trabalho e AF doméstica. Além disso, considerando que se trata de estudo transversal, a causalidade reversa pode limitar a força dos resultados.

## Conclusão

De acordo com os resultados observados no presente estudo, sugere-se que a prática da AF discrimina e está associada a níveis mais elevados de HDL-C em adultos de ambos os sexos. É importante ressaltar que, nos homens, existe a necessidade de maior quantidade de atividades físicas (duração e intensidade) para que os benefícios possam ser alcançados. Esses resultados são de grande importância para a saúde pública, considerando que as políticas públicas de promoção de atividades físicas podem ser norteadas com base nessas informações. Sugerem-se estudos longitudinais sobre o tema para que se possa confirmar os presentes resultados.
