# Supplementary material for: Associação entre Atividade Física no Tempo Livre e HDL-C em Participantes do Elsa-Brasil: Existem Diferenças entre Homens e Mulheres no Efeito Dose-Resposta?
Source: Arq Bras Cardiol. 2021 Jun 25;117(3):494–500. [Article in Portuguese] doi: 10.36660/abc.20200438 (PMC8462941; doi:10.36660/abc.20200438)
Supplement: Translation

# Translation: Association between Leisure Time Physical Activity and HDL-C in the Elsa-Brasil Study Participants: Are There Any Gender Differences in the Dose-Response Effect?

**Authors:** Francisco José Gondim Pitanga, Rosane Harter Griep, Maria da Conceição Almeida, Maria de Jesus Mendes da Fonseca, Andreia Rios de Souza, Raiane de Carvalho Silva, Sheila Maria Alvim Matos

**Affiliations:** 1 Brasil Programa de Pós-Graduação em Ciências da Reabilitação - Universidade Federal da Bahia, Salvador, BA - Brasil; 2 Brasil Laboratório de Educação em Meio Ambiente e Saúde, Instituto Oswaldo Cruz, Fiocruz, Rio de Janeiro, RJ - Brasil; 3 Brasil Instituto Gonçalo Moniz, Salvador, BA - Brasil; 4 Brasil Escola Nacional de Saúde Pública (Fiocruz), Rio de Janeiro, RJ - Brasil; 5 Brasil Universidade Federal da Bahia, Salvador, BA - Brasil; 6 Brasil Instituto de Saúde Coletiva - Universidade Federal da Bahia, Salvador, BA - Brasil

**Keywords:** Cardiovascular Diseases, Exercise, Physical Activity, Cross-Sectional Studies, HDL-Cholesterol, Epidemiology, Walking

## Abstract

**Background:**

High levels of high-density lipoprotein (HDL-C) are known for their protective effect against cardiovascular diseases and the regular practice of leisure time physical activity (LTPA) may be associated with their increase.

**Objective:**

To verify the existence of differences between genders in the dose-response effect regarding the association between LTPA and HDL-C in the ELSA-Brasil study cohort.

**Methods:**

Cross-sectional study with data from wave 2 of 13,931 participants of both genders (7,607 women) from the Longitudinal Study of Adult Health ELSA-Brasil. The LTPA was measured using the International Physical Activity Questionnaire (IPAQ) and classified into four categories: sedentary, low active, active and very active. The discriminatory power of LTPA at different intensities analyzed for high HDL-C was tested using ROC curves. Associations, adjusted for confounders between LTPA and HDL-C were analyzed by logistic regression. A 95% confidence interval was used.

**Results:**

A positive association, with a dose-response effect, was observed between LTPA and HDL-C in both men and women. With regard to intensity, only vigorous physical activity discriminated high HDL-C in men, while both walking and moderate and vigorous physical activity discriminated high HDL-C in women.

**Conclusions:**

LTPA shows a positive association with gradient dose-response and HDL-C, but in men, the association is not observed for those classified as physically unfit. In women, both walking intensity and moderate or vigorous physical activity can discriminate high HDL-C levels, whereas only vigorous intensity-exercise discriminate elevated HDL-C levels in men, demonstrating that males need to do more physical activity for this benefit to be observed.

## Introduction

The regular practice of physical activity (PA) shows a positive association with the increase in high density lipoprotein (HDL-C) and decrease in triglyceride levels, with inconsistent changes in low-density lipoprotein levels.^1^ In addition, higher levels of HDL-C may have benefits such as protecting against coronary artery disease, although more recent Mendelian randomization studies have suggested that HDL-C is more of a marker of cardiovascular risk than a risk factor itself.^2 , 3^

The increase of HDL-C associated with PA practice may be explained to the extent that, during PA, there is an increase in lipoprotein lipase activity (LPL), accelerating the breakdown of triglyceride-rich lipoprotein. This generates the transfer of cholesterol, phospholipids and proteins to the nascent particles of HDL-C.^4^ However, in more physically active individuals, the HDL-C half-life may reach 6-7 days, while in the physically inactive, it is only 3-4.^5^

Despite this evidence, the ideal PA intensity to increase HDL-C needs to be better clarified, mainly with emphasis in possible differences between men and women as shown in previous studies about PA and cardiometabolic health.^6^

In a recent research carried out in participants of the Longitudinal Study of Adult Health ELSA-Brasil, the results show that there is an association between PA and higher HDL-C levels, both in men and women. Moreover, it was observed that vigorous-intensity PA practice is associated with more positive changes in lipid profiles, than only the duration of PA practice, However, this study did not analyze possible differences between men and women about the quantities (time and duration) of PA.^7^

The knowledge of existence of a dose-response effect between PA and HDL-C increase will contribute to the production of new information and researches on the subject, considering that public policies for PA promotion may use this information as a basis for new intervention proposals. Moreover, although the association between PA and HDL-C levels is already known, studies that demonstrate the dose-response effect in relation the possible differences between men and women are scarce in assessed literature about the subject. Studies that reaffirm the association between PA and HDL-C are still important for better understanding of this association, mainly in the analysis of possible differences between the genders regarding these findings.

The objective of study was to verify the existence of differences between the genders in the dose-response effect regarding the association between LTPA and HDL-C in the ELSA-Brasil cohort

## Methods

### Population and sample

The ELSA-Brasil is a cohort study of 15,105 active or retired civil servants aged 25 to 74 years from six higher education institutions located in the cities of Salvador, Vitória, Belo Horizonte, Rio de Janeiro, São Paulo e Porto Alegre, which has as its main objective to investigate the incidence and progression of diabetes and cardiovascular diseases and their associated factors (biological, behavioral, environmental, occupational, psychological and social), whose methodological details were previously described.^8 , 9^ To date, information has been collected about participants in three opportunities: (1^st^wave, from 2008 to 2010); (2^nd^wave, from 2012 to 2014) and (3^rd^wave, from 2016 to 2018). For the present study, was selected all participants of the second wave (2012-2014) that answered questionnaires about PA and had complete data on the variables involved in the analysis, totaling 13,931 participants (7,607 women).

The ELSA-Brasil was approved by the National Research Ethics Commission (CONEP) and by all Research Ethics Committees of the six research centers involved. All participants signed the informed consent form and the confidentiality of the data is guaranteed.

### Data production

The data were collected by a team of interviewers and raters trained and certified by a quality control committee,^9^ able to perform the study protocol in any ELSA-Brasil Research Center. Face-to-face interviews were conducted when applying the blocks of questionnaires to obtain information on age, level of schooling, family income and smoking. Additionally, information on anthropometric measurements of weight, height and waist circumference was collected. Body weight was obtained in the morning, after 8-12 hours of fasting and with the participant without shoes and wearing light clothes. An electronic Toledo®scale was used to measure weight, with a capacity of up to 200 Kg. A SECA® stadiometer was used to measure height, with the participant in the standing position and strictly following the Frankfurt plan. Obesity was identified through the body mass index (BMI) by applying the equation BMI= weight (Kg)/height(m)^2^adopting the following cut-off points; obesity = 0 if BMI < 30.0 and obesity = 1 if BMI ≥30.0.

### Evaluation of Physical Activity

The long version of the International Physical Activity Questionnaire (IPAQ) was used for the identification and qualification of PA, which consists of questions related to the frequency and duration of physical activities (walking, moderate PA and vigorous PA) developed at work, during commuting, in domestic activities and leisure time.^10^ Only leisure time and commuting domains were assessed in the ELSA-Brasil. The PA was measured in minutes/week by multiplying the weekly frequency by the duration of each of the performed activities. For the study effect,, only leisure-time physical activity (LTPA) was utilized, with the following categorization: 0= physically inactive (less than 10 minutes of any PA in the week); 1= unfit (from 10 minutes to less than 150 minutes of walking/moderate activity per week and/or from 10 minutes to less than 60 minutes of vigorous activity for week and/or from 10 minutes to less than 150 minutes per week of any combination of walking, moderate and vigorous activity); 2= physically active ( more than or equal to 150 minutes of walking/moderate activity per week and/or more than or equal to 60 minutes of vigorous activity per week and/or more than or equal to 150 minutes per week of any combination of walking/ moderate and vigorous activity ); 3= very active (more than or equal to 150 minutes of vigorous activity per week, or more than or equal to 60 minutes of vigorous activity per week + 150 minutes per week of any combination between walking and moderate activity). For the dichotomized analyses, those classified as physically inactive and unfit were considered as insufficiently active, and those classified as physically active and very active as active.

### Evaluation of HDL-C

Blood collection was performed after 12 hours of overnight fasting and centrifuged and stored in cryo-tubes at -80°C in the respective research centers. To ensure the quality and standardization of the results, all samples were sent for processing and analysis in a central ELSA-Brasil laboratory located in the city of São Paulo. The HDL-C values in mg/dL were determined by a homogeneous colorimetric method using an ADVIA 1200 Siemens equipment.^7^ HDL-C was dichotomized at 60 mg/dL and ≥ 60 mg/dL (high HDL-C).

### Covariates

The following categorization was adopted for the covariates: age=0 if between 34 and 50 years, age=1 if between 51 and 60 years and age=2 if >60years. Four strata were established for the level of schooling: 0= incomplete elementary school; 1=complete elementary school; 2= complete high school; and 3= complete higher education / postgraduation. The family income was categorized as: up to 2 minimum wages=0; from 2 to 8=minimum wages=1; from 8 to 18 minimum wages=2; and above 18 minimum wages=3. The current smoking status was categorized as no=0 and yes=1.

### Data analysis

The descriptive measures (proportions) were calculated for all categorized variables. The analysis was stratified by gender *a priori* and compared using the chi-square test. ROC (Receiver Operating Characteristic) curves were used to test the discriminatory power of different LTPA intensities (walking, moderate PA and vigorous PA) for elevated HDL-C levels.^11^ The 95% confidence interval (95%CI) of was utilized. The associations were analyzed by logistic regression. The following variables were considered as potential confounders or effect modifiers: age, obesity, smoking at the moment of the interview, income family and level of schooling. The variables that showed p ≤ 0.05 and correlation coefficient Rho <0.60 in the simultaneous evaluation in the bivariate stage (tetrachoric matrix) were selected for modeling.

The verification of the effect modification was performed by stratification with the observation of stratum-specific point measures and their confidence interval. Effect modification. was indicated when the point measurement of a factor, in a specific stratum, was not contained in the confidence interval of the other factor in the same stratum. The analysis of possible confounding variables was performed using the backward procedure, using logistic regression. The analysis begins with the complete model, followed by removal of the potential confounders one by one. When a change equal to or greater than 10% in the point association between LTPA and HDL-C is observed, the variable is considered to be a confounder.^12^ No effect-modifying variables were identified in the modeling process, and the age variable was identified as a confounder. Therefore, the best model for the analysis of an association between LTPA and HDL-C was the age-adjusted model. The dose-response effect also analyzed the association between LTPA and HDL-C. For this analysis, variables called DUMMIES were created for comparison between the reference group (physically inactive) and each of the other strata of the PA variable (insufficiently active, active and very active). The Mantel-Haenszel test was used to test the homogeneity of the odds ratio (OR) values between the strata of each variable with a significance level of 5%. The confidence interval was set at 95%. The statistical program STATA® version 12.0 was used.

## Results

A total of 6,324 men and 7,607 women was included in the analysis. The sample characteristics are shown in table 1 . The women’s mean age is 55,7±8.8 and the men’s is 55,7±9.2. It is also observed that men have higher family income, are more often smokers, are more active in leisure time, while the women are more obese and have a higher proportion of higher HDL-C levels. It is also observed that there are no statistically significant differences between men and women in relation to age.

Table 2 shows the discriminatory power of the different intensities of LTPA for the increase in HDL-C levels. Among men, only the vigorous PA showed statically significant areas under the ROC curves, while among women, both walking and moderate and vigorous physical activity showed statistical significance to discriminate the increase in HDL-C levels, although the areas under the ROC curves did not show high values.

**Table 2:** – Areas under the ROC curves for different physical activity intensities as a discriminator for high HDL-C (≥60 mg/dL)

Leisure Time Physical Activity	Male	Female
Walking	0.52 (0.50-0.54)	0.52 (0.51-0.53) *
Moderate	0.52 (0.50-0.54)	0.53 (0.53-0.54)*
Vigorous	0.54 (0.52-0.56)*	0.53 (0.52-0.54)*

ROC: receiver operating characteristic; 95% CI: confidence interval of 95%. *Area under the ROC curve with discriminatory power IM (Li-IC > 0.50). )

The association between LTPA and HDL-C in male and female individuals is shown in Table 3 . It can be observed that active men have a 38% chance of having higher HDL-C levels, while in active women the chance is 41%, compared to physically inactive men and women, respectively.

**Table 3:** – Association between leisure time physical activity and HDL-C

Leisure time physical activity	Male	Female
Insufficiently Active (Physically Inactive and Little Active) Active (Active and Very Active)	1.00 1.38 (1.20-1.59)	1.00 1.41 (1.29-1.55)

* Adjusted by age

Table 4 shows that when the PA was classified into 4 categories, there was a dose-response effect in the association between LTPA and HDL-C, both in men and women. However, in male individuals, the association was found only in those classified as active and very physically active.

**Table 4:** – Dose-response effect in the association between leisure time physical activity and HDL-C

Leisure time physical activity	Male	Female
Inactive Little Actives Active Very Active	1.00 1.21 (0.98-1.50) 1.27 (1.05-1.52) 1.77 (1.46-2.15)	1.00 1.15 (1.01-1.31) 1.33 (1.19-1.49) 1.81 (1.56-2.09)

* Adjusted by age.

## Discussion

The study analyzed the existence of a dose-response effect in the association between LTPA and HDL-C increase.

Is important to point out that the study population is a cohort of civil servant volunteers, which although are not representative of the general population, represent a significant number of participants in six Brazilian capitals, a fact that embodies a strong point of the study.

The mechanisms that associate higher PA intensity and more positive lipid changes are explained by the fact that during the PA there is an increase in LPL activity and triglyceride catabolism, which results in the transfer of cholesterol, phospholipids and proteins to the nascent HDL-C particles, thus increasing its concentration.^4 , 5^ In addition, the average half-life of HDL-C particles is higher in more physically active individuals, a fact that can contribute to the maximization of the reverse transport of cholesterol.^5^

In this context, in a study carried out with participants of ELSA-Brasil, it was demonstrated that there is a beneficial association between PA levels and a favorable lipid profile for HDL-C and triglycerides, both in men and women. Moreover, it was also observed that the higher the intensity of PA, the more positive the changes in lipid profile.^7^

In another study carried out with individuals living in the municipality of Caxias do Sul, state of Rio Grande do Sul, Brazil, who were over 60 years old, it was shown that those who performed physical activities of moderate intensity had reduced LDL-C and increased HDL-C levels, when compared to older adults who had low PA^13^ levels.

According to Matsudo et al.,^14^ there is little evidence regarding the mechanics responsible for the reduction of LDL-C and very low-density lipoprotein (VLDL-C) levels, but the main reason for the increase in HDL-C levels is the greater action of LPL in response to physical exercise, a fact that increase the catabolism of triglycerides and causes the transfer of cholesterol, phospholipids and proteins to HDL-C particles, thus increasing its levels.^14^ Other studies have shown that physically active men and women have higher HDL-C levels and lower levels of LDL-C and VLDL-C than their physically inactive pairs.^15^ Moreover, another study conducted only with female individuals, it was observed that there are significant differences in HDL-C levels in women with high, moderate and low levels of physical fitness, and the highest values were found in women with high levels of physical fitness.^16^

It is also important to emphasize that in our study, the intensity of LTPA was classified as walking; moderate PA; and vigorous PA. In this context, although the areas under the ROC curves do no show high values, only the vigorous PA discriminates high HDL-C in men, while in women, both the walking and moderate or vigorous PA discriminate high HDL-C levels, demonstrating that men need more intense PA to increase HDL-C levels.

Another important aspect to be highlighted in this study is that the dose-response effect between LTPA and HDL-C has significance both in women classified as little active and in women classified as active and very active. In men, the association is observed only in those classified as active and very active. Therefore, it is possible to understand that they need to practice a greater amount of PA to have a positive result regarding the HDL-C increase.

In a recent publication, also with data form the ELSA-Brasil, aiming to verify the association between LTPA and PA during commuting with cardiovascular risk scores, it was observed that only LTPA was inversely associated with cardiovascular events; moreover, the men needed a greater amount of PA (duration and intensity), also with a dose-response effect, than women to reach the benefits.^17^

These results can be partially explained by the fact that, in men, the resting homeostatic parameters, such as heart rate, blood pressure, glycemic levels, caloric expenditure, among others, are higher than in women, although in the postmenopausal period and with the aging process, blood pressure values may be higher in the female sex.^18 , 19^ Thus, to disrupt the resting homeostasis, the men need a greater amount of PA (duration and intensity) than women, to activate the mechanisms that trigger cardiovascular protection, such as the lowering of blood pressure, reduction of glycemia and triglyceride levels, and increase in HDL-C levels (which at rest show lower levels in men).^6^

A possible limitation can be attributed to the information on PA, since it was obtained through questionnaires, which, despite being a widely used instrument in national and international studies, may present problems, such as memory bias. In addition, is important to note that only LTPA was analyzed in the present study, and other domains of PA were not considered, such as commuting, PA at work and domestic PA. Moreover, considering this is a cross-sectional study, reverse causality may limit the strength of the results.

## Conclusions

According to the results observed in this present study, it is suggested that the practice of PA discriminates and is associated with higher levels of HDL-C in adults of both genders. It is important to emphasize that men need a greater amount of physical activities (duration and intensity) for benefits to be achieved. These results are of great importance for public health, considering that public policies for the promotion of physical activities can be guided based on this information. Longitudinal studies on the subject are suggested to confirm the present results.

**Table 1:** – Characteristics of the study sample

	Men (n=6324)	Women (n=7607)	p
**Age (years) - n (%)** 34-50 51-60 >60	2055 (32.5) 2182 (34.5) 2087 (33.0)	2342 (30.8) 2752 (36.2) 2511 (33.0)	0.49
**Family income (minimum wages) - n (%)** Up to 2 From 2 to 8 From 8 to 18 More than 18	60 (0.9) 2117 (33.6) 2462 (39.1) 1657 (26.3)	84 (1.1) 2545 (33.6) 3356 (44.3) 1583 (20.9)	<0.01
**Education - n (%)** Incomplete Elementary School Complete Elementary School Complete High School C omplete Higher Education/ Postgraduation	448 (10.2) 482 (10.9) 1933 (43.9) 1533 (34.9)	261 (4.5) 390 (6.7) 2449 (42.3) 2694 (46.)	<0.01
**Obesity - n (%)** BMI < 30 kg/m^2^ BMI ≥30 kg/m^2^	4763 (75.3) 1561 (24.7)	5308 (69.8) 22299 (30.2)	<0.01
**Current smoking– n (%)** No Yes **Leisure time physical Activity - n (%)** Physically Inactive Insufficiently Active Active Very Active **High HDL-C levels** Yes No	3290 (52.0) 3033 (48.0) 2218 (35.1) 1089 (17.2) 1749 (27.6) 1268 (20.1) 916 (14.5) 5408 (85.5)	4863 (63.9) 2744 (36.1) 3401 (44.7) 1342 (17.6) 1947 (25.6) 917 (12.0) 3.241 (42.7) 4.366 (57.3)	<0.01 <0.01 <0.01

*Values for men and women were compared using the chi-square test.*
